# Viperin suppresses goose astrovirus type 2 replication through inhibition of viral RdRP activity

**DOI:** 10.1186/s13567-026-01817-8

**Published:** 2026-07-30

**Authors:** Haoran Xu, Jinhang Gao, Junjie Yang, Wenwen Cheng, Yan Cheng, Yingjun Lv

**Affiliations:** College of Veterinary Medicine, Nanjing Agricultural University, Nanjing, 210095 China

**Keywords:** Goose astrovirus 2, Viperin, RdRp activity, ddhCTP, viral replication, goslings

## Abstract

Goose astrovirus 2 (GAstV-2) infection induces visceral gout in goslings, resulting in high mortality rates in birds aged 3–15 days. Despite extensive research since the virus was first identified in 2018, the innate immune defense mechanisms of the host against GAstV-2 remain incompletely characterized, impeding the development of effective vaccines and therapeutics. Here, we demonstrated that GAstV-2 infection upregulated Viperin expression both in vitro and in vivo, suggesting a potential role for Viperin as a host restriction factor. Further studies revealed that the P2 domain of viral ORF2 contributed to stimulate Viperin. Overexpression and knockdown experiments confirmed that Viperin exerts antiviral activity against GAstV-2. However, Viperin did not affect attachment or internalization. Mechanistic studies revealed that Viperin inhibits GAstV-2 by attenuating viral RNA-dependent RNA polymerase (RdRp) activity in a manner dependent on its S-adenosylmethionine (SAM) domain. The SAM domain was found to promote the production of 3'-deoxy-3',4'-didehydro-cytidine-5'-triphosphate (ddhCTP), which correlated with reduced RdRp activity and suppression of viral replication. Furthermore, intramuscular administration of ddhCTP reduced mortality and viral loads, and ameliorated GAstV-2-induced pathological injury in the kidney, spleen, and liver of goslings. Collectively, these findings identify Viperin as a key host restriction factor against GAstV-2 and highlight its potential as a target for antiviral strategies.

## Introduction

Goose astrovirus 2 (GAstV-2) is a nonenveloped, positive-sense, single-stranded RNA virus with a genome approximately 7.2 kb in length. The viral genome comprises a 5′-untranslated region (5′-UTR), three open reading frames (ORF1a, ORF1b, and ORF2), and a 3′-untranslated region (3′-UTR). ORF1a and ORF1b encode nonstructural proteins, including RNA-dependent RNA polymerase (RdRp), a serine protease motif (Pro), and a nuclear localization signal (NLS), which are primarily involved in viral RNA replication and transcription. ORF2 encodes the capsid (Cap) protein, which plays a key role in the host immune response. GAstV-2 predominantly infects goslings aged between 3 and 15 days of age, with mortality rates ranging from 5% to 50%. Infected birds typically exhibit visceral and articular urate deposition, renal swelling, and hepatic vacuolar degeneration and necrosis [1–4]. Currently, no commercially available drugs or vaccines exist for the control of GAstV-2, leading to substantial economic losses in the goose breeding industry.

At present, most isolated GAstV-2 strains have been shown to induce no cytopathic effect (CPE) and produce low virus titers in Leghorn Male Hepatoma cell line (LMH) cells, which currently represent the only cell line supporting GAstV-2 replication [5]. Consequently, GAstV-2 infection is considered self-limiting, and cellular innate immunity is thought to play a critical role in suppressing or restricting viral replication and spread. Interferons (IFNs) serve as the first line of defense in innate immunity and are essential for controlling viral invasion by activating hundreds of interferon-stimulated genes (ISGs), the products of which exert direct antiviral effects [6]. Viral nucleic acids are recognized by pattern recognition receptors (PRRs), such as Toll-like receptor 3 (TLR3), RIG-1, and MDA-5, triggering IFN production that subsequently induces ISG expression via the Janus kinase/signal transducers and activators of transcription (JAK/STAT) signaling pathway [7, 8]. Previous studies have reported that GAstV-2 infection upregulates PRRs including TLR3, RIG-1, and MDA-5, and elevates the expression of antiviral cytokines such as IFN-α, IFN-β, MX, IFITM3, and OASL in the kidneys and spleens of infected goslings [9, 10]. Moreover, overexpression of OASL in LMH cells has been shown to restrict GAstV-2 replication [5]. Besides OASL, however, the function of other antiviral proteins in GAstV-2 replication remain unclear.

Our previous RNA sequencing analysis revealed that GAstV-2 infection induced high-level expression of Viperin in primary goose kidney tubular epithelial cells [6]. Viperin, also known as radical S-adenosyl methionine domain 2 (RSAD2), belongs to the radical S-adenosylmethionine (SAM) enzyme superfamily [11, 12]. It is an evolutionarily conserved type I ISG with well-documented antiviral activity against several viruses, such as West Nile virus (WNV), dengue virus (DENV), and human immunodeficiency virus (HIV) [13–18]. However, Viperin does not exert antiviral effects against all viruses. For instance, it has been shown to promote HCMV infection and DNA replication in a manner dependent on its iron-sulfur cluster [19, 20]. Whether Viperin inhibits GAstV-2 replication and the underlying molecular mechanisms is still not clear.

In this study, we demonstrated that GAstV-2 infection upregulates Viperin expression both in vitro and in vivo. Overexpression of Viperin was found to restrict GAstV-2 replication. Mechanistically, the SAM domain of Viperin is crucial for the antiviral activity, as it facilitates the production of 3'-deoxy-3',4'-didehydro-cytidine-5'-triphosphate (ddhCTP), which in turn inhibits viral RdRp activity. Consistent with this, the addition of ddhCTP suppressed both viral RdRp activity and viral replication in vitro. Furthermore, intramuscular injection of ddhCTP reduced mortality and viral loads in the kidney of goslings infected with GAstV-2. Collectively, these findings identify Viperin as a key host defense factor against GAstV-2 infection and reveal a potential cellular target for developing antiviral strategies against GAstV-2.

## Materials and methods

### Cells and virus

LMH cells were maintained in our laboratory and cultured in DMEM/F-12 (Gibco, Life Technologies, USA) supplemented with 10% fetal bovine serum (FBS, Bio-Channel, China) and 1% penicillin (100 U/ml)-streptomycin (10 mg/mL) at 37 °C with 5% CO_2_. Goose renal tubular epithelial (GRTE) cells were isolated and cultured according to our previous study [6]. GAstV-2 stain JSHA (GenBank: MK125058, 10^4.25^ TCID_50_ /mL) used in this study was isolated and preserved in our laboratory [21].

### Plasmid construction and transfection

The full-length coding sequences of Viperin (Flag-tagged), GAstV-2 ORF1a, ORF1b, ORF2, S, P1 and P2 (HA-tagged), and the Viperin mutants (Viperin Δ1–44, Viperin Δ250–354, and Viperin DCA) were cloned into the pcDNA3.1( +) eukaryotic expression vector. All primers used for plasmids construction are listed in Table 1. Linearized vector and corresponding polymerase chain reaction (PCR) fragments were assembled using the ClonExpress II One Step Cloning Kit (Vazyme, Nanjing, China). Transient transfection of cells was carried out using Lipofectamine 3000 reagent (Thermo Fisher, USA).

**Table 1:** **Primers used for plasmids construction**

Primers	Primers sequences (5′–3′)
Viperin-F	CTAGCGTTTAAACTTAAGCTTGCCACCATGCTGCTGGGCGTTCT
Viperin-R	TGCTGGATATCTGCAGAATTCTTACTTATCGTCGTCATCCTTGTAATCCCAGTCCAGAATCATGTCTGCTTTACT
Viperin Δ1-44-F	CTAGCGTTTAAACTTAAGCTTGCCACCATGCGGTCGGGCCC
Viperin Δ250-354-R	TGCTGGATATCTGCAGAATTCTTACTTATCGTCGTCATCCTTGTAATCCCAGTCCAGAATCATGTCTGCTTTACT
Viperin-DCA-F	AAGGCGGGTTTCGCGTTCCACACGGCCAAGACCT
Viperin-DCA-R	GAACGCGAAACCCGCCTTGTAGTTGCACTGCCTGGTG
ORF1a-F	CTAGCGTTTAAACTTAAGCTTGCCACCATGGCGGCCGGTGG
ORF1a-R	TGCTGGATATCTGCAGAATTCCTAAGCGTAATCTGGAACATCGTATGGGTAGTTTTTTTCACACGTTTCACGAGTCACG
ORF1b-F	CTAGCGTTTAAACTTAAGCTTGCCACCATGAAAAAACTAGATAAGGGGGACGATGC
ORF1b-R	TGCTGGATATCTGCAGAATTCTCAAGCGTAATCTGGAACATCGTATGGGTACCAGATTTGAGAAAAGAAGTCGGGC
ORF2-F	CTAGCGTTTAAACTTAAGCTTGCCACCATGCTGCCTCTGCCAGTG
ORF2-R	TGCTGGATATCTGCAGAATTCTCAAGCGTAATCTGGAACATCGTATGGGTACTCATGTCCGCCCTTCTCAAAG
ORF2-S-F	CTAGCGTTTAAACTTAAGCTTGCCACCATGGCAGACAGGGCGGTG
ORF2-S-R	TGCTGGATATCTGCAGAATTCTCAAGCGTAATCTGGAACATCGTATGGGTACCCTGGCTTTGGACCATAATTCGA
ORF2-P1-F	CTAGCGTTTAAACTTAAGCTTGCCACCATGCTCTCACTCATGACCAGTGAAA
ORF2-P1-R	TGCTGGATATCTGCAGAATTCTCAAGCGTAATCTGGAACATCGTATGGGTAACCTTGTCCAGTTGTATTCACATTTGG
ORF2-P2-F	CTAGCGTTTAAACTTAAGCTTGCCACCATGCTGCCTCTGCCAGTG
ORF2-P2-R	TGCTGGATATCTGCAGAATTCTCAAGCGTAATCTGGAACATCGTATGGGTAAGAGGTCTTGAGCGAGACTGC

### GAstV-2 absorption, internalization, and replication assay

For the absorption assays, LMH cells cultured in 12-well plates or 24-well plates were transfected with pViperin-Flag for 24 h at 37℃. After three washes with cold phosphate-buffered saline (PBS), cells were inoculated with GAstV-2 at multiplicity of infection (MOI) of 1 at 4 °C for 1 h. The inoculum was removed, the cells were washed three times with cold PBS before harvesting.

For the internalization assay, LMH cells were transfected with pViperin-Flag for 24 h at 37 °C, and after three washes with cold PBS, the cells were inoculated with GAstV-2 at MOI of 1 at 4 °C for 1 h. The inoculum was removed, and the cells were washed three times with cold PBS. The cells were further incubated with medium for 1 h at 37 °C, and then the cells were washed three times with PBS and subsequently treated with 0.01% trypsin for 5–10 min at 37 °C to inactivate and remove noninternalized viruses.

For replication assay, cells were infected with GAstV-2 at MOI of 0.01 at 37 °C at 24 h post-transfection. After the incubation period, the cells were washed with PBS, followed by incubation in medium containing 2% FBS at 37 °C. Viral loads at 24 and 48 hpi were determined using a SYBR Green I-based real-time PCR method as previously established [22].

### Quantitative real-time PCR (qPCR)

Total RNA was extracted from infected cells using the RNA-easy Isolation Reagent (Vazyme, Nanjing, China) and was reverse-transcribed into cDNA using HiScript II Q RT SuperMix for qPCR (Vazyme, Nanjing, China) following the manufacturer’s instructions. Quantitative real-time PCR was performed using ChamQ SYBR Color qPCR Master Mix (Vazyme, Nanjing, China) on a CFX96 thermal cycler (Bio-Rad, USA). The primers used for qPCR analysis will be made available upon request. Target gene mRNA expression levels were quantified via the 2^−ΔΔCT^ method and normalized to *GAPDH* expression.

### Western blot

Cells were lysed with RIPA buffer supplemented with 1 × Protease Inhibitor Cocktail (MCE, Shanghai, China) and protein concentrations were quantified using the bicinchoninic acid (BCA) assay. Equal amounts of protein samples were mixed with 5 × SDS loading buffer and heated at 99 °C for 10 min. Mouse anti-capsid monoclonal antibody (generated in house), rabbit anti-Viperin antibody (Proteintech Group, China), mouse anti-GAPDH antibody (Abcam), and mouse-anti-Flag antibody (Beyotime, Beijing, China) were used as primary antibodies, and Horseradish peroxidase (HRP)-conjugated goat anti-mouse immunoglobulin G (IgG) or goat anti-rabbit IgG (Abclonal, Wuhan, China) served as the secondary antibody. Sodium dodecyl sulfate-polyacrylamide gel electrophoresis (SDS-PAGE) was performed as previously described [23]. After membrane incubation with Clarity Western ECL Blotting Substrate, protein bands were visualized using the ChemiDoc Touch Imaging System (Bio-Rad, Hercules, CA). Target protein expression levels were then normalized to GAPDH and analyzed using Image J software.

### siRNA interference

Three distinct Viperin-specific short interfering (si)RNAs were designed and synthesized (Table 2). LMH cells were transfected with each siRNA or a negative control siRNA using Lipofectamine 3000 (Invitrogen), following the manufacturer’s instructions. The transfected cells were then subjected to subsequent experimental analyses.

**Table 2:** **Sequences of the siRNAs used in the study**

Primers	Primers sequences (5′–3′)
Negative control	Sense (5′–3′): ACGUGACACGUUCGGAGAATT
	Antisense (5′–3′): UUCUCCGAACGUGUCACGUTT
siViperin-1	Sense (5′–3′): CCAGGCAGUGCAACUACAATT
	Antisense (5′–3′): UUGUAGUUGCACUGCCUGGTT
siViperin-2	Sense (5′–3′): GGUUCAAGAAGUAUGGUGATT
	Antisense (5′–3′): UCACCAUACUUCUUGAACCTT
siViperin-3	Sense (5′–3′): CCGUGAUCAACAGAUUUAATT
	Antisense (5′–3′): UUAAAUCUGUUGAUCACGGTT

### Cell-based virus RdRp activity assay

LMH cells were cultured in 24-well plates and transfected with the respective plasmids. Cells were then treated with or without indicated concentrations of NTPs, followed by transfection with the GAstV-2 RdRp activity assay system. After 20 h, firefly luciferase (Fluc) and Renilla luciferase (RLuc) activities were measured using the TransDetect^®^ Double-Luciferase Reporter Assay Kit (TransGen Biotech, Beijing, China). RLuc values were normalized to FLuc values for data analysis.

### ddhCTP detection in cell lysates

LMH cells transfected with different plasmids were harvested and lysed with cell lysis buffer (TransGen Biotech, Beijing, China). After centrifugation at 12,000 × *g* for 12 min at 4 °C, the supernatant was ultrafiltered using a 3-kDa cutoff Amicon Ultra-0.5 centrifugal filter unit (Merck) at 14,000 × *g* for 20 min at 4 °C. The flow-through fraction containing molecules below 3 kDa was collected as the lysate sample for ddhCTP quantification by liquid chromatography–mass spectrometry (LC–MS). LC–MS analysis was performed on a Sciex Triple Quad 6500 system. Prior to analysis, 10 µL of each sample was mixed with 40 µL of an acetonitrile: methanol mixture (5:3, v/v). The mixture was vortexed thoroughly, centrifuged at 17,000 × *g* for 2 min, and 3 µL of the resulting supernatant was injected onto an EVO C18 column (2.6 µm, 2.1 × 20 mm). The mobile phase comprised 5 mM DMHA in water (solvent A) and 5 mM DMHA in acetonitrile (solvent B). Chromatographic separation was performed at a constant flow rate of 0.3 mL/min under the following gradient conditions: 5% solvent B held on for 3 min, followed by a linear gradient from 5% to 30% solvent B over 5 min.

### Animal experiments

Before the experiments, five 1-day-old goslings were euthanized by intravenous injection of sodium pentobarbital. No gross changes were found in all organs, and GAstV-2 RNA was not detected by PCR method. Then, 30 1-day-old unvaccinated goslings were randomly assigned to 3 groups: the control group, GAstV-2 group, and ddhCTP (5 mg/d) group, with 10 goslings in each group. Each group of goslings was separated by a negative-pressure isolator and provided with sterilized water and antibiotic-free feed ad libitum. Except the control group, all 4-day-old goslings were orally challenged with 0.5 mL GAstV-2 (0.5 × 10^4.25^TCID_50_/goose) according to our previous studies [24, 25]. In the control group, 0.5 mL PBS was orally administered. For the next 5 days, goslings in the control and GAstV-2 groups were muscularly injected daily with 0.3 mL PBS, and goslings in the ddhCTP group were muscularly injected daily with 0.3 mL of 1.5 mM ddhCTP (Tianjin Olivebio Biotechnology Co., Ltd). All geese were monitored daily for the occurrence of clinical signs. Dead goslings were dissected, and pathological changes were recorded immediately. At 7 d post infection (dpi), all goslings were weighed, and blood samples were taken from the wing vein; then, the goslings were euthanized by intravenous injection of pentobarbital sodium. The kidneys were collected and weighed, and the relative weights of the kidney was calculated as the ratio of kidney weight to gosling weight. A portion of the kidneys was fixed in 4% paraformaldehyde for histopathological examination. The rest of the kidney tissue was stored at −80 °C for further analysis.

### Histopathological examination

Kidney samples were fixed in 4% paraformaldehyde, dehydrated through a graded series of alcohols, cleared with xylene, and embedded in paraffin. The embedded tissues were then serially sectioned at a thickness of 4 μm. The sections were stained with hematoxylin and eosin (HE) following routine protocols, and subsequently examined under a light microscope (Carl Zeiss, Göttingen, Germany).

### Viral RNA and cap protein detection in kidney

Viral RNA was extracted from kidney samples using the RNeasy Isolation Kit (Vazyme, Nanjing, China) and reverse-transcribed into cDNA with the HiScript Q RT SuperMix Kit (Vazyme, Nanjing, China). Viral loads were then determined by a SYBR Green I-based real-time PCR method established in our previous study [22]. For protein analysis, kidney tissues were lysed in RIPA buffer supplemented with 1 × Protease Inhibitor Cocktail (MCE, Shanghai, China), and protein concentrations were quantified using the BCA assay. Cap protein expression was subsequently measured as described above (Section Western blot).

### Immunohistochemical analysis

Kidney sections were deparaffinized, rehydrated, treated with 3% H_2_O_2_ to block endogenous peroxidase, and subjected to antigen retrieval in citrate buffer at 100 °C. After blocking with 5% bovine serum albumin, sections were incubated overnight at 4 °C with a mouse anti-GAstV-2 Capsid monoclonal antibody, followed by secondary antibody incubation at 37 °C for 1 h. Diaminobenzidine staining and hematoxylin counterstaining were then performed. Positive signals were semiquantitatively analyzed using Image-Pro Plus software.

### Statistical analysis

The differences between different groups were analyzed by *t*-test using GraphPad Prism 8.0 software. Data are presented as means ± standard deviations (SDs). Significance levels were set as follows: *P < *0.05*; **P < *0.01*.

## Results

### GAstV-2 infection induces robust Viperin expression in vitro and in vivo

**Figure 1:**
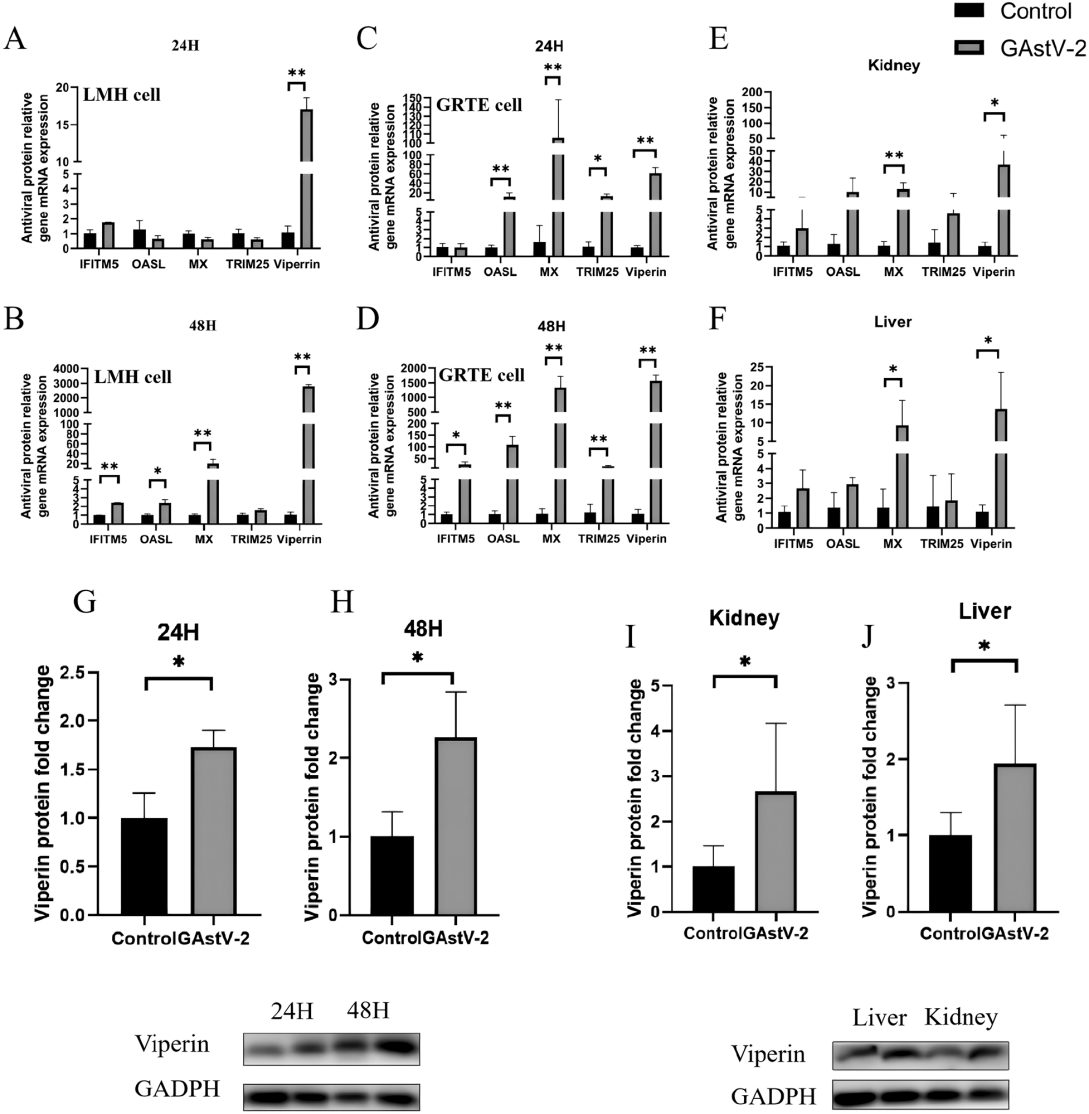
**GAstV-2 activates innate immune responses in vitro and in vivo.** **A**, **B** Transcript levels of innate immune-related genes (IFITM5, OASL, MX, TRIM25, and Viperin) in GAstV-2-infected and control LMH cells at 24 and 48 hpi. **C**, **D** Transcript levels of innate immune-related genes in GAstV-2-infected and control GRTE cells at 24 and 48 hpi. **E**, **F** Transcript levels of innate immune-related genes in the kidney (**E**) and liver (**F**) from GAstV-2-infected and control goslings. **G**, **H** Protein levels of Viperin in GAstV-2-infected and control LMH cells at 24 and 48 hpi. **I**, **J** Protein levels of Viperin in the kidney (**I**) and liver (**J**) from GAstV-2-infected and control goslings. In panels **A**–**D**, **G**, and **H**, data are presented as mean ± SD from three biological replicates; in **E**, **F**, **I**, and **J**, data are presented as mean ± SD from five gosling samples. Student’s *t*-test was used for comparison between control and GAstV-2 groups (*P < *0.05*, **P < *0.01*).

To identify host antiviral factors potentially responsive to GAstV-2 infection, LMH cells and GRTE cells were infected with GAstV-2 at MOI of 1, and the gene expression levels of antiviral proteins including IFITM5, OASL, MX, TRIM25, and Viperin were assessed at 24 and 48 h post-infection (hpi). Results showed that GAstV-2 infection upregulated mRNA expression of IFITM5, OASL, MX, and Viperin in LMH cells, and all five antiviral proteins in GRET cells (Figure 1A–D) (*P* < 0.05). Among these, Viperin exhibited the most pronounced upregulation. Gene expression was also analyzed in kidney and liver tissues from our previous gosling infection study [6]. As shown in Figure 1E, F, mRNA levels of Viperin and MX were obviously elevated in infected goslings compared with controls (*P* < *0.05*). To further validate Viperin induction at the protein level, Western blot analysis was performed. Consistent with the mRNA data, GAstV-2 infection resulted in marked higher Viperin protein expression in LMH cells, kidney, and liver compared with controls (Figure 1G–J) (*P* < *0.05*). Taken together, these findings demonstrate that GAstV-2 infection strongly induces Viperin expression both in vitro and in vivo.

### ORF2 plays a vital role in activating Viperin expression

**Figure 2:**
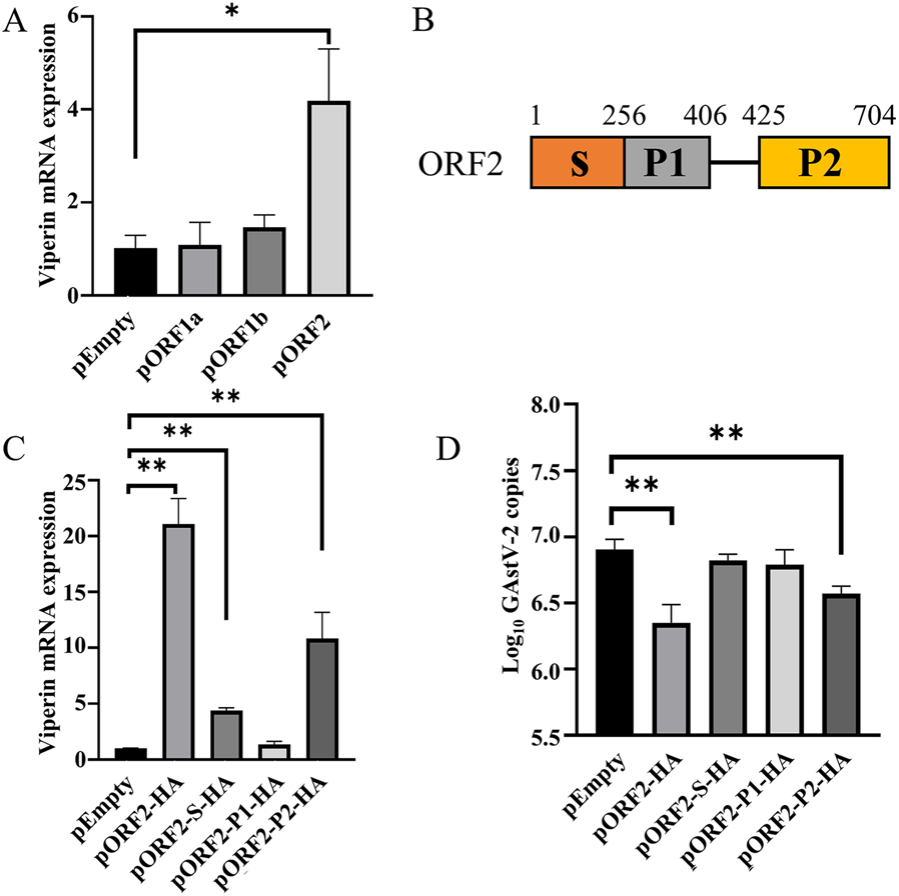
**GAstV-2 ORF2 plays a vital role in activating Viperin expression. A** LMH cells were transfected with plasmids expressing ORF1a, ORF1b, or ORF2 for 48 h. Total RNA was then extracted and Viperin mRNA expression was analyzed by qRT-PCR. **B** Schematic representation of recombinant plasmids encoding different truncated ORF2 constructs. **C** LMH cells were transfected with plasmids expressing full-length ORF2 or the truncated domains S, P1, and P2 for 48 h, after which Viperin mRNA expression was quantified by qRT-PCR. **D** LMH cells were transfected with the indicated plasmids and then infected with GAstV-2 at MOI of 0.01. Viral copy numbers were measured by qRT-PCR at 48 hpi. In **A**, **C**, and **D**, data are presented as mean ± SD from three biological replicates. Student’s *t*-test was used to compare the empty plasmid group with the recombinant plasmid groups (*P < *0.05*, **P < *0.01*).

To identify which component of GAstV-2 is responsible for inducing high levels of Viperin, three plasmids encoding viral open reading frames (ORF1a, ORF1b, and ORF2) were constructed and transfected into LMH cells. As shown in Figure 2A, ORF2 significantly upregulated Viperin expression compared with the control (*P* < *0.05*), whereas ORF1a and ORF1b did not. To further determine which domain of ORF2 is essential for Viperin activation, three ORF2 truncations were constructed on the basis of a previous study [5] (Figure 2B). Among these, the truncated P2 domain markedly induced Viperin expression and suppressed viral replication (Figure 2C and D). These results indicate that the P2 domain effectively activates Viperin production.

### Viperin efficiently inhibits GAstV-2 replication

**Figure 3:**
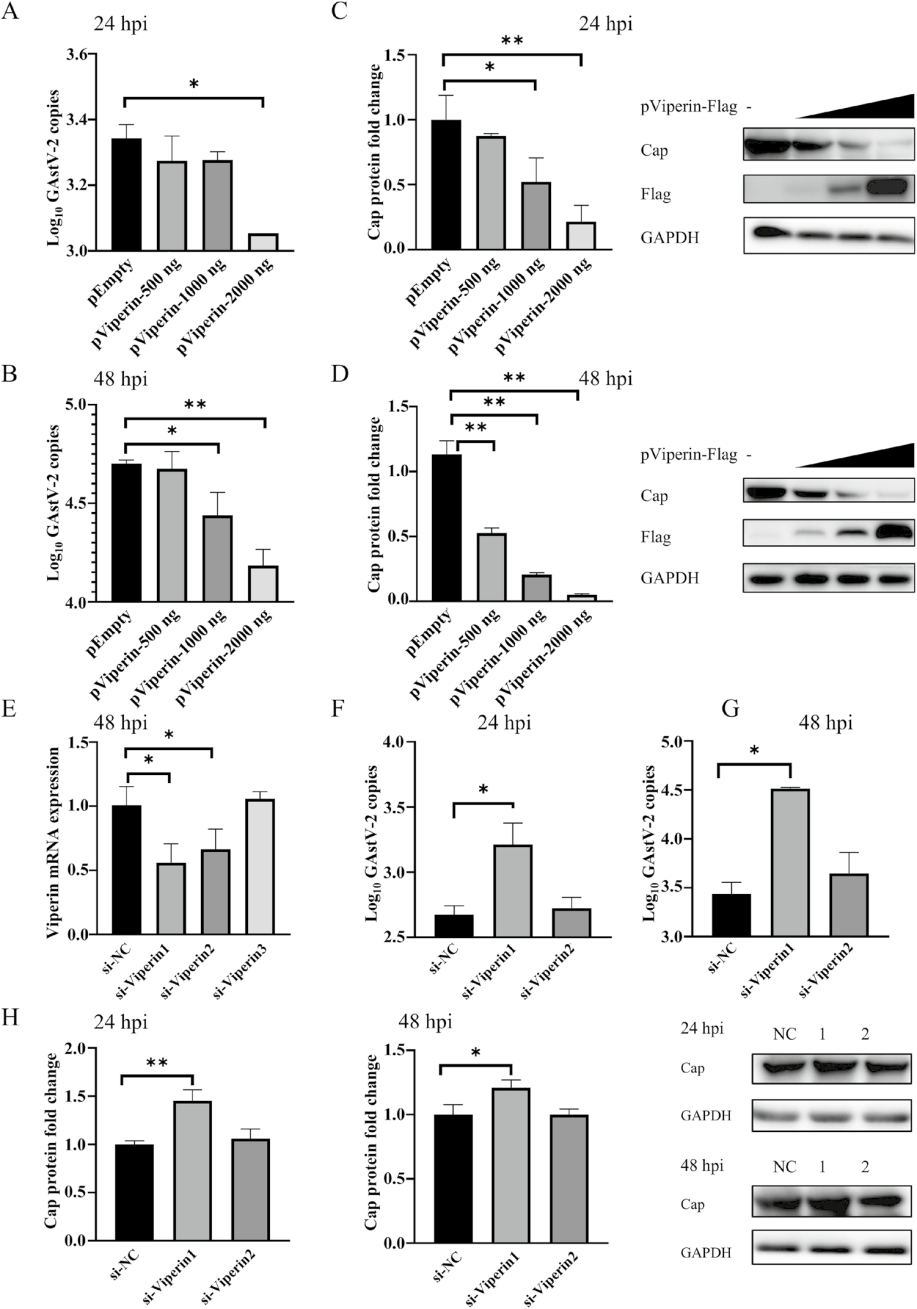
**Viperin inhibits GAstV-2 infection in vitro.** **A**, **B** Viral load in LMH cells transfected with increasing doses of pViperin followed by GAstV-2 infection (MOI = 0.01) were measured by qRT-PCR at 24 hpi (**A**) and 48 hpi (**B**). **C**, **D** Western blot analysis of GAstV-2 capsid protein expression in transfected and infected LMH cells at 24 hpi (**C**) and 48 hpi (**D**). **E** Efficiency of Viperin knockdown in LMH cells transfected with three distinct Viperin-specific siRNAs, measured by qRT-PCR at 48 hpi. **F**, **G** Viral loads in LMH cells transfected with siViperin-1 or siViperin-2 followed by GAstV-2 infection (MOI = 0.01), measured by qRT-PCR at 24 hpi (**F**) and 48 hpi (**G**). **H** Western blot analysis of GAstV-2 capsid protein levels in siRNA-transfected and virus-infected LMH cells at 24 and 48 hpi. In **A**–**H**, data are presented as mean ± SD from three biological replicates. Student’s *t*-test was used for comparison: in **A**–**D**, empty plasmid group versus increasing doses of the Viperin plasmid; in **E**–**H**, negative control siRNA group versus Viperin-specific siRNA groups (*P < *0.05*, **P < *0.01*).

To assess the antiviral role of Viperin against GAstV-2, LMH cells were transfected with either an empty vector or a Viperin-expressing plasmid, followed by infection with GAstV-2 at MOI of 0.01. Cells were harvested at indicated timepoints post-infection and analyzed using qRT-PCR and Western blot. Compared with the control, both viral RNA copies and capsid protein levels were significantly reduced in Viperin-overexpressing cells at 24 and 48 hpi (*P* < *0.05*), with the inhibitory effect exhibiting dose dependence (Figure 3A–D). To further examine Viperin function, three Viperin-specific siRNAs were designed to knock down endogenous Viperin expression in LMH cells. As shown in Figure 3E, both si-Viperin1 and si-Viperin2 significantly reduced Viperin mRNA levels (*P* < *0.05*), whereas si-Viperin3 showed no notable effect (*P* > *0.05*), indicating effective knockdown by si-Viperin1 and si-Viperin2. When LMH cells transfected with si-Viperin1 or si-Viperin2 were infected with GAstV-2 (MOI = 0.01) and analyzed at 24 h and 48 h, both viral copies and capsid protein levels were significantly increased in si-Viperin1-transfected cells relative to the control (*P* < *0.05*) (Figure 3F–H). Collectively, these results demonstrate that Viperin act as a potent host restriction factor that suppresses GAstV-2 replication.

### Viperin inhibits GAstV-2 infection at a post-entry step

**Figure 4:**
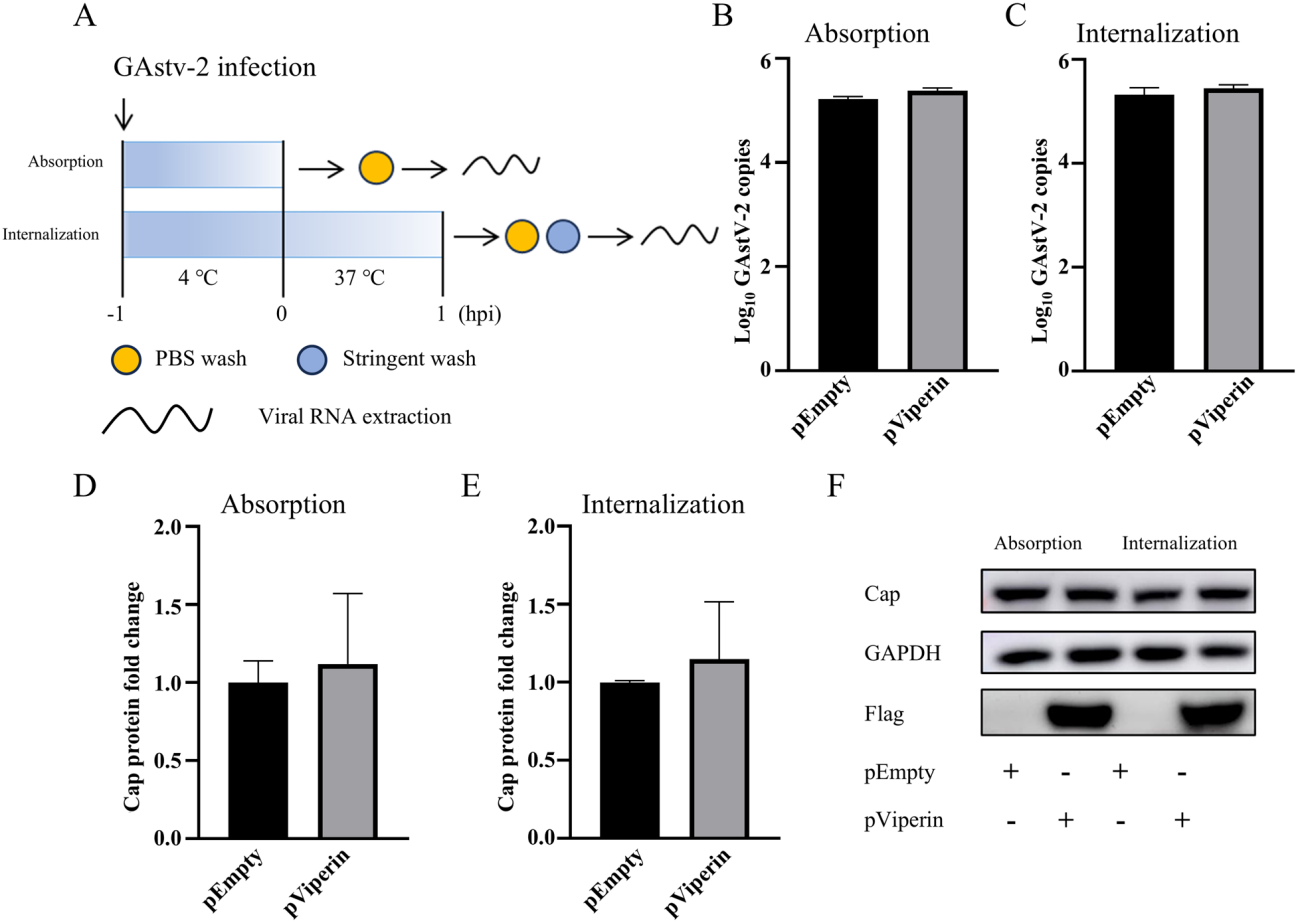
**Viperin restricts GAstV-2 replication at a post-entry stage.** **A** Schematic diagram of the experimental design for assessing viral adsorption and internalization. **B** Viral adsorption analysis: LMH cells with or without Viperin overexpression were incubated with GAstV-2 (MOI = 1) at 4 °C for 1 h. Cell-associated viral RNA copies were quantified by qRT-PCR. **C** Viral internalization analysis: following adsorption at 4 °C, Viperin-overexpressing LMH cells were shifted to 37 °C for 1 h to allow viral entry, treated with 0.01% trypsin to remove noninternalized virus, and intracellular viral RNA copies were measured by qRT-PCR. (**D**–**F**) Protein-level analysis: GAstV-2 capsid protein levels were detected by Western blot under adsorption (**D**, **F**) and internalization (**E**, **F**) conditions, corresponding to the experimental schemes in **B** and **C**, respectively. In **B**–**F**, data are presented as mean ± SD from three biological replicates. Student’s *t*-test was used to compare the empty plasmid group with the Viperin plasmid group.

To determine which stage of the GAstV-2 replication cycle is affected by Viperin, we examined viral absorption and internalization using the experimental scheme outlined in Figure 4A. For the absorption experiment, LMH cells, with or without Viperin overexpression, were incubated with GAstV-2 at MOI of 1 for 1 h at 4 °C, thoroughly washed with PBS, and bound viral genome copies were quantified by qRT-PCR. For the internalization experiment, Viperin-overexpressing LMH cells were incubated with GAstV-2 (MOI = 1, 1 h, 4 °C) and then shifted to 37 °C for 1 h to permit internalization. Cells were then treated with 0.01% trypsin and washed to remove unbound and noninternalized virus. Intracellular viral RNA copies and capsid protein were then detected by qRT-PCR and western blot, respectively. Results showed no significant differences in the amounts of absorbed or internalized virus between vector control and Viperin transfected cells (Figure 4B–F), suggesting that Viperin overexpression does not block the process of viral binding and entry.

### Viperin inhibits GAstV-2 replication by reducing viral RdRp activity

**Figure 5:**
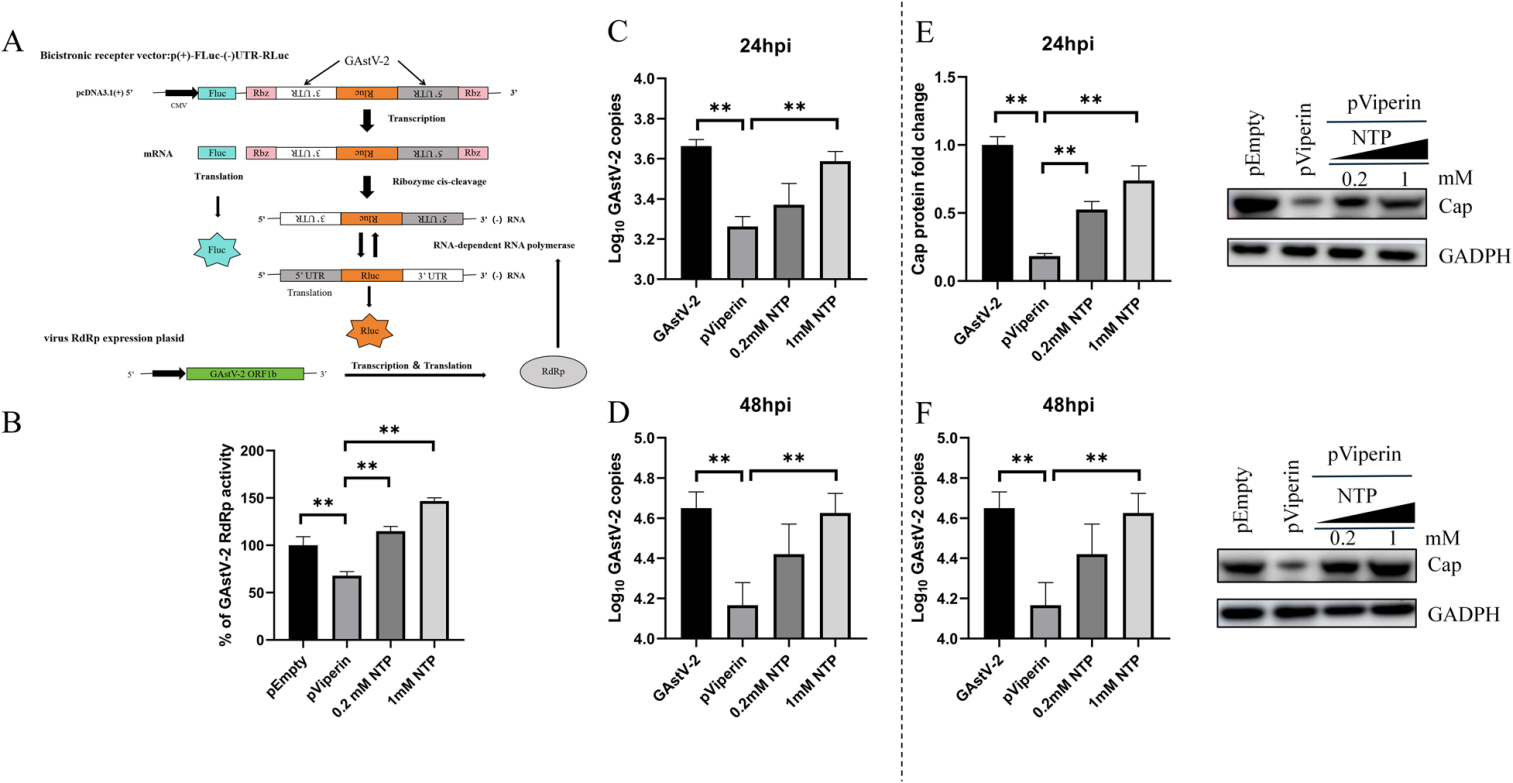
**Viperin suppresses GAstV-2 RdRp activity.** **A** Schematic representation of the cell-based reporter assay system for evaluating GAstV-2 RdRp activity. **B** RdRp activity in LMH cells transfected with pViperin or empty vector for 24 h, followed by GAstV-2 infection (MOI = 0.01) and treatment with varying NTP concentrations, as measured by the reporter assay system. **C**, **D** Viral load in transfected and infected LMH cells under different NTP conditions at 24 hpi (**C**) and 48 hpi (**D**), quantified by qRT-PCR. **E**, **F** GAstV-2 capsid protein expression in transfected and infected LMH cells at 24 hpi (**E**) and 48 hpi (**F**), detected by Western blot analysis. In **B**–**F**, data are presented as mean ± SD from three biological replicates. Student’s *t*-test was used to compare the empty plasmid group with the treated groups (*P < *0.05*, **P < *0.01*).

GAstV-2 depends on its RdRp for replication. Previous studies have shown that Viperin converts CTP to ddhCTP, a nucleotide analog that acts as a chain terminator for RdRp, thereby inhibiting the replication of several RNA viruses. To examine whether Viperin impairs GAstV-2 replication through inhibition of RdRp activity, we established a GAstV-2-specific RdRp activity assay on the basis of a previously described system [26]. The bicistronic reporter plasmid contains a sense-oriented firefly luciferase gene [( +)FLuc] under the control of a CMV promoter (serving as an internal normalization control), and an antisense-oriented Renilla luciferase sequence [( −)RLuc] flanked by hepatitis delta virus (HDV) ribozyme self-cleavage sites, as well as antisense GAstV-2 5′ and 3′ UTRs. Following host transcription of the full-length ( +) FLuc-( −) UTR-RLuc RNA, autocatalytic cleavage at HDV ribozyme sites releases an antisense 3′UTR-( −) RLuc-5′UTR transcript. This RNA serves as a substrate for GAstV-2 RdRp during infection, and Renilla luciferase activity quantitatively reflects RdRp activity (Figure 5A). Using this assay, we evaluated the effect of Viperin on GAstV-2 RdRp activity. As shown in Figure 5B, Viperin overexpression significantly reduced the RLuc/FLuc ratio—a measure of RdRp activity (*P* < *0.01*). In contrast, exogenous NTP supplementation restored RdRp activity in a dose-dependent manner. Consistent with this, the addition of NTPs reversed the Viperin-mediated reduction in viral RNA copies and capsid protein expression at both 24 and 48 hpi (Figure 5C–F). Together, these results indicate that Viperin restricts GAstV-2 replication by diminishing RdRp activity.

### Viperin suppresses GAstV-2 replication through ddhCTP production

**Figure 6:**
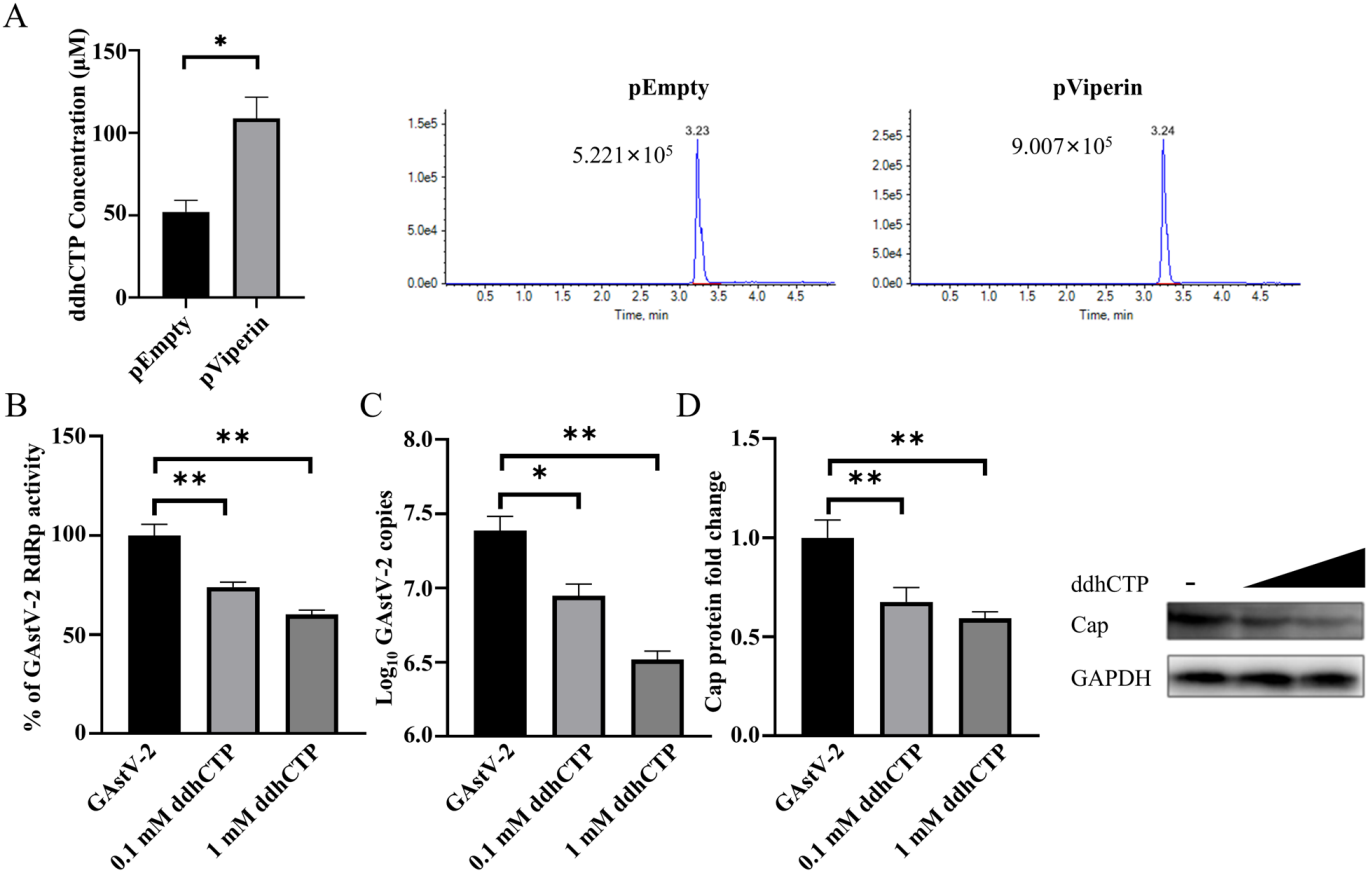
**Viperin restricts GAstV-2 replication through ddhCTP production.** **A** Quantification of intracellular ddhCTP levels in LMH cells following transfection with pViperin or empty vector control for 24 h, as determined by LC–MS analysis. **B** GAstV-2 RdRp activity in ddhCTP-treated and GAstV-2-infected LMH cells using a cell-based reporter assay system. **C** Viral load in ddhCTP-treated and GAstV-2-infected LMH cells at 36 hpi, measured by qRT-PCR at 36 hpi. **D** GAstV-2 capsid protein expression in ddhCTP-treated and GAstV-2-infected LMH cells at 36 hpi, detected by WB at 36 hpi. In **A**–**D**, data are presented as mean ± SD from three biological replicates. Student’s *t*-test was used for comparison: in **A**, empty plasmid group versus Viperin plasmid group; in **B**–**D**, GAstV-2-infected group versus ddhCTP-treated groups (*P < *0.05*, **P < *0.01*).

As mentioned above, Viperin catalyzes the conversion of CTP to ddhCTP, which serves as a chain terminator for RdRp. To assess the production and antiviral function of ddhCTP in the context of GAstV-2 infection, we measured intracellular ddhCTP levels in Viperin-overexpressing LMH cells. As shown in Figure 6A, Viperin overexpression significantly increased ddhCTP accumulation compared with the control (*P* < *0.05*). Exogenous addition of ddhCTP reduced GAstV-2 RdRp activity in a dose-dependent manner (Figure 6B). Consistent with this, ddhCTP supplementation also led to dose-dependent decreases in viral RNA copies and capsid protein expression in GAstV-2-infected LMH cell (Figure 6C, D). Collectively, these findings suggest that Viperin induced ddhCTP biosynthesis, which in turn inhibits decreased GAstV-2 replication by suppressing RdRp activity.

### The radical SAM domain is essential for Viperin-mediated restriction of GAstV-2 replication

**Figure 7:**
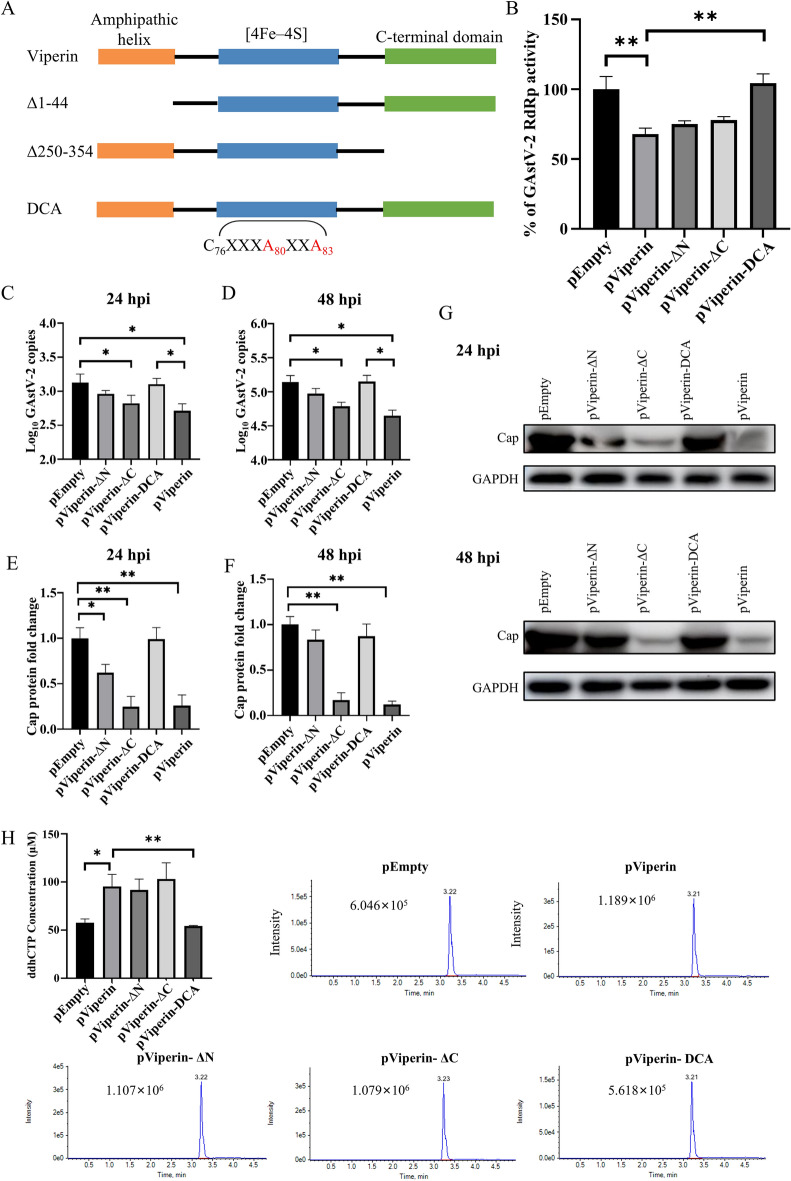
**The radical SAM domain is essential for Viperin's antiviral function.** **A** Schematic representation of Viperin domain architecture and mutant constructs. **B** GAstV-2 RdRp activity in LMH cells transfected with wild-type Viperin, Viperin mutants (ΔN, ΔC, DCA), or empty vector for 24 h, followed by infection with GAstV-2 (MOI = 0.01), assessed using a cell-based reporter assay. **C**, **D** Viral loads in transfected and infected LMH cells at 24 hpi (**C**) and 48 hpi (**D**), quantified by qRT-PCR. **E**–**G** GAstV-2 capsid protein expression in transfected and infected LMH cells at 24 hpi (**E**, **G**) and 48 hpi (**F**, **G**), measured by WB. **H** Intracellular ddhCTP concentrations in transfected and infected LMH cells, measured by LC–MS. In **B**–**G**, data are presented as mean ± SD from three biological replicates. Student’s *t*-test was used to compare the Viperin plasmid group with the empty plasmid group and with the Viperin mutant plasmid groups (*P < *0.05*, **P < *0.01*).

To identify the functional domain of Viperin required for GAstV-2 replication, we constructed three mutant plasmids: Viperin Δ1-44 (lacking the N-terminal domain), Viperin Δ250-354 (lacking the C-terminal domain), and Viperin DCA (a radical SAM domain mutant with C_80_A/C_83_A substitutions that disrupt the [4Fe-4S] cluster) (Figure 7A). When tested in the GAstV-2 RdRp activity assay, the SAM domain mutant (DCA) significantly restored RdRp activity that had been suppressed by Viperin (*P* < *0.05*), whereas neither N- nor C-terminal truncation had a notable effect (*P* > *0.05*) (Figure 7B). We next evaluated the impact of these mutations on viral replication. As shown in Figure 7C–G, both N-terminal deletion and the SAM domain mutant significantly increased the viral RNA copies and Cap protein levels compared with the Viperin group at 24 and 48 hpi (*P* < *0.05*), whereas the C-terminal truncation did not restore viral replication (*P* > *0.05*). To determine whether these effects correlated with ddhCTP production, we measured intracellular ddhCTP levels in cells expressing each mutant. The SAM domain mutant significantly reduced ddhCTP accumulation relative to the Viperin group (*P* < *0.05*), while neither terminal truncation affected ddhCTP levels (Figure 7H). Collectively, these results demonstrate that the radical SAM domain activity is essential for Viperin’s antiviral function, and that its enzymatic activity drives ddhCTP production to limit GAstV-2 replication.

### ddhCTP suppresses viral replication and mitigates GAstV-2-induced pathology in goslings

**Figure 8:**
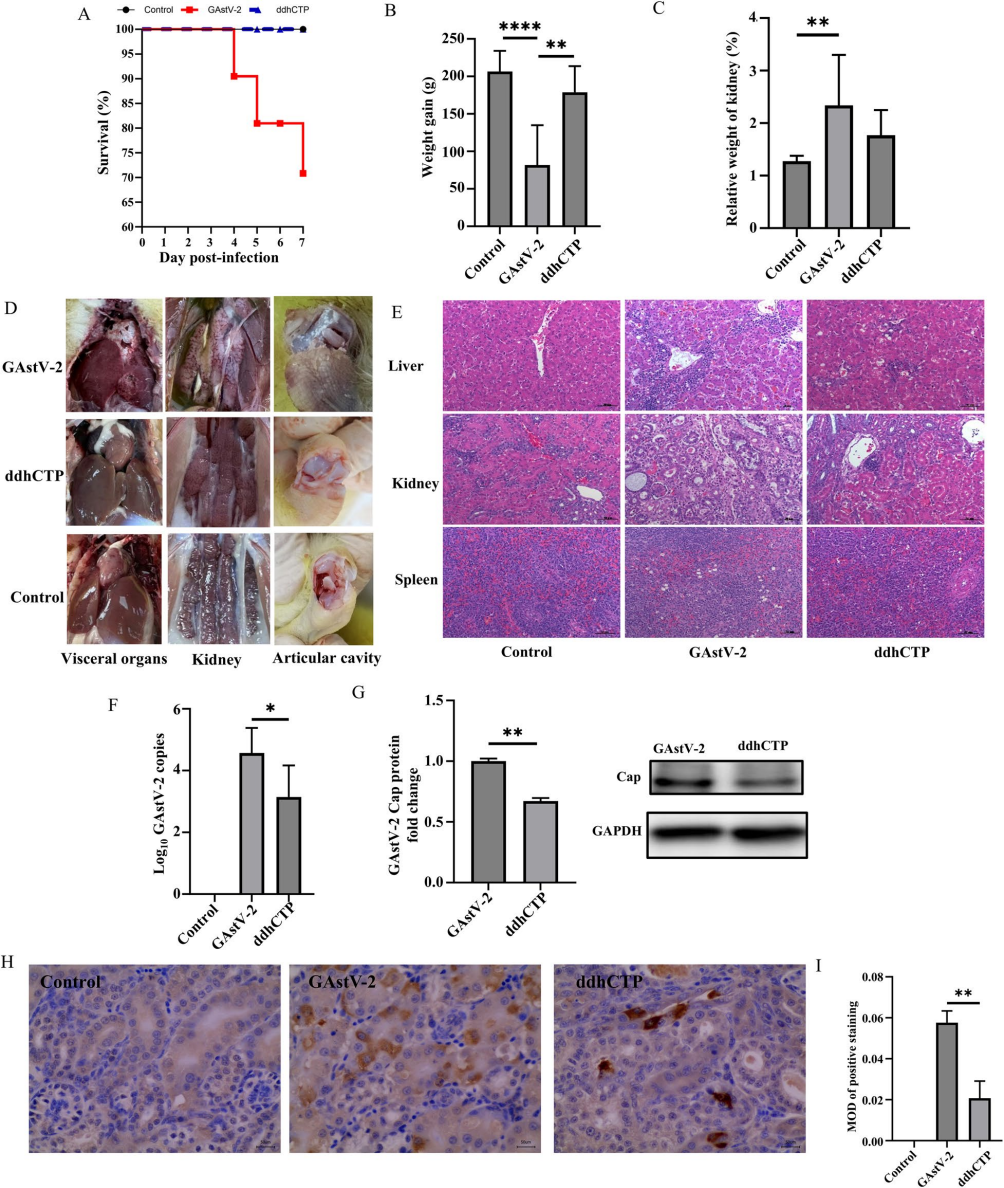
**ddhCTP administration ameliorates GAstV-2 pathogenesis in goslings.** **A** Survival rates of GAstV-2-infected goslings following intramuscular ddhCTP administration. **B** Body weight gain in infected goslings treated with ddhCTP or vehicle control at 7 dpi. **C** Relative kidney weight in different experimental groups at 7 dpi. **D** Representative gross morphological changes in different treatment groups. **E** Histopathological analysis of kidney, liver, and spleen sections from infected goslings with or without ddhCTP treatment. **F** Viral loads in kidney tissues quantified by qRT-PCR. **G** GAstV-2 capsid protein expression in kidney tissues detected by Western blot analysis. **H** The virus location was detected by immunohistochemistry and the mean optical density (MOD) of positive staining was calculated using Image-Pro Plus (**I**). In **B**, **C**, **F**, **G**, and **I**, data are presented as mean ± SD from ten gosling samples. Student’s *t*-test was used to compare the GAstV-2 group with the control group and with the ddhCTP group (*P < *0.05*, **P < *0.01*).

To evaluate the therapeutic potential of ddhCTP against GAstV-2 infection in vivo, goslings were challenged with GAstV-2 and administered ddhCTP intramuscularly for three consecutive days. GAstV-2 infection resulted in 30% mortality and significantly reduced body weight gain compared with controls (Figure 8A, B). In contrast, no mortality was observed in the ddhCTP-treated group, and body weight gain was significantly improved relative to the GAstV-2-infected group (*P* < *0.05*). Post-mortem examination of dead goslings revealed extensive urate deposition in the liver, kidneys, and joint cavities. Moreover, most GAstV-2-infected goslings exhibited renal swelling or hemorrhage, accompanied by an increase in relative kidney weight. Conversely, ddhCTP treatment markedly alleviated renal lesions, reduced the relative kidney weight, and prevented urate deposition in visceral organs and joint cavities (Figure 8C, D). Histopathological examination indicated that GAstV-2 infection was associated with significant tissue damage, primarily characterized by perivascular inflammation in the liver, tubular necrosis with interstitial inflammation, and fibrosis in the kidney, and marked splenic lymphocyte depletion. Importantly, treatment with ddhCTP effectively ameliorated these specific lesions in all affected tissues (Figure 8E). Consistent with these findings, the viral load, Cap protein expression, and virus distribution in kidney tissues were all significantly lower in the ddhCTP-treated group than in the GAstV-2 infection group (*P* < *0.05*) (Figure 8F–I). Collectively, these results indicate that intramuscular administration of ddhCTP reduces mortality, decreases viral replication, and alleviates tissue damage in GAstV-2-infected goslings.

## Discussion

Since its initial isolation in 2018, GAstV-2 has remained a major pathogen threatening the goose industry. The limited understanding of GAstV-2–host interactions has hindered the development of effective drugs and vaccines. Furthermore, the low viral titers observed in goose embryos and cell cultures, along with the absence of cytopathic effects in many GAstV-2 isolates, suggest a finely balanced host–virus relationship. Interferons (IFNs) serve as the first line of defense in innate immunity, inducing the expression of numerous antiviral proteins. In this study, we evaluated the expression of several antiviral genes, including IFITM5, OASL, MX, TRIM25, and Viperin–in LMH cells, GRTE cells, and tissues of GAstV-2-infected goslings. Among these, Viperin exhibited the most pronounced upregulation, suggesting a particularly significant role in the host response to GAstV-2 infection.

Viperin, a member of the radical S-adenosylmethionine (SAM) enzyme superfamily, is widely recognized for its potent and broad-spectrum antiviral activity across diverse species and cell types. This multifunctional protein functions as an antiviral ribonucleoside synthase, modulates secretion and lipid raft organization, regulates β-oxidation and thermogenesis, and enhances antiviral signaling pathways [11, 12]. Through overexpression and knockdown experiments in LMH cells, we demonstrated that Viperin significantly restricts GAstV-2 replication, confirming its role as a host restriction factor during infection. Structurally, Viperin contains an N-terminal domain with an amphipathic α-helix and leucine zipper motif, a central radical SAM domain, and a highly conserved C-terminal domain [27, 28]. Our domain-mutation analyses revealed that disruption of the SAM domain most substantially restored GAstV-2 replication, followed by N-terminal deletion, whereas C-terminal deletion had no significant effect, highlighting the essential role of the SAM domain in Viperin-mediated inhibition of GAstV-2. These findings align with prior studies demonstrating that SAM mutations impair Viperin’s antiviral activity against West Nile virus (WNV), dengue virus (DENV), and human immunodeficiency virus (HIV) [13–18]. Mechanistically, Viperin catalyzes the conversion of CTP to ddhCTP via a SAM-dependent radical mechanism [29]; ddhCTP acts as a chain-terminating ribonucleotide analog that inhibits viral RdRp activity. Consistent with this model, Viperin overexpression in LMH cells increased ddhCTP levels and suppressed GAstV-2 RdRp activity, whereas SAM mutation reduced ddhCTP production and restored RdRp activity. Although N-terminal deletion did not affect ddhCTP synthesis, it reduced viral copy number and Cap protein expression, indicating that the N-terminal domain also contributes to Viperin’s antiviral activity, possibly through mechanisms independent of radical SAM activity. The N-terminal amphipathic α-helix is known to anchor Viperin to the endoplasmic reticulum and lipid droplets, localizations critical for its antiviral function [12, 30]; the precise antiviral mechanism of the N-terminal region in the context of GAstV-2 infection warrants further investigation.

ddhCTP, a recently identified antiviral nucleotide, acts as a chain terminator of RdRp-catalyzed RNA synthesis in multiple RNA viruses, including DENV, WNV, and Zika virus (ZIKV) [29], though it is ineffective against others such as influenza A virus, poliovirus, and human rhinovirus [29, 31]. Here, we showed that ddhCTP inhibits GAstV-2 replication in LMH cells and reduces clinical symptoms and mortality in infected goslings. Given the current lack of approved veterinary treatments for gosling gout caused by GAstV-2, ddhCTP—or compounds that induce its endogenous production—represents a promising candidate for reducing disease-related losses.

However, this study has certain limitations. Although we found that ORF2, particularly the P2 domain, induces Viperin expression, the underlying mechanism was not investigated. Previous studies have revealed a complex regulatory network governing Viperin expression. Viperin expression is primarily regulated by type I (IFN-α/β) and type II (IFN-γ) interferons via the JAK/STAT signaling pathway, in which STAT1/STAT2 heterodimers recruit interferon regulatory factors (IRFs) to initiate Viperin transcription [32, 33]. In hepatitis C virus infection, hepatic sinusoidal endothelial cells (LSECs) significantly upregulate Viperin expression through IFN-α/β autocrine signaling, thereby inhibiting viral replication [34]. In addition, Viperin is regulated by the TLR signaling pathway. For example, TLR4 activates Viperin expression via its downstream NF-κB and IRF3 pathways in response to various bacterial and viral infections [35]. Moreover, Viperin can be induced independently of the interferon pathway, directly regulated by IRF1 or IRF3 [36, 37]. Vesicular stomatitis virus infection stimulates IRF1 to bind to the two proximal ISREs of the Viperin promoter, thereby activating its transcription [38]. Viperin expression can also be directly induced by activation of MAVS and downstream IRF3 following chikungunya virus infection [39]. The specific mechanism by which GAstV-2 induces Viperin production warrants further investigation.

## Conclusions

**Figure 9:**
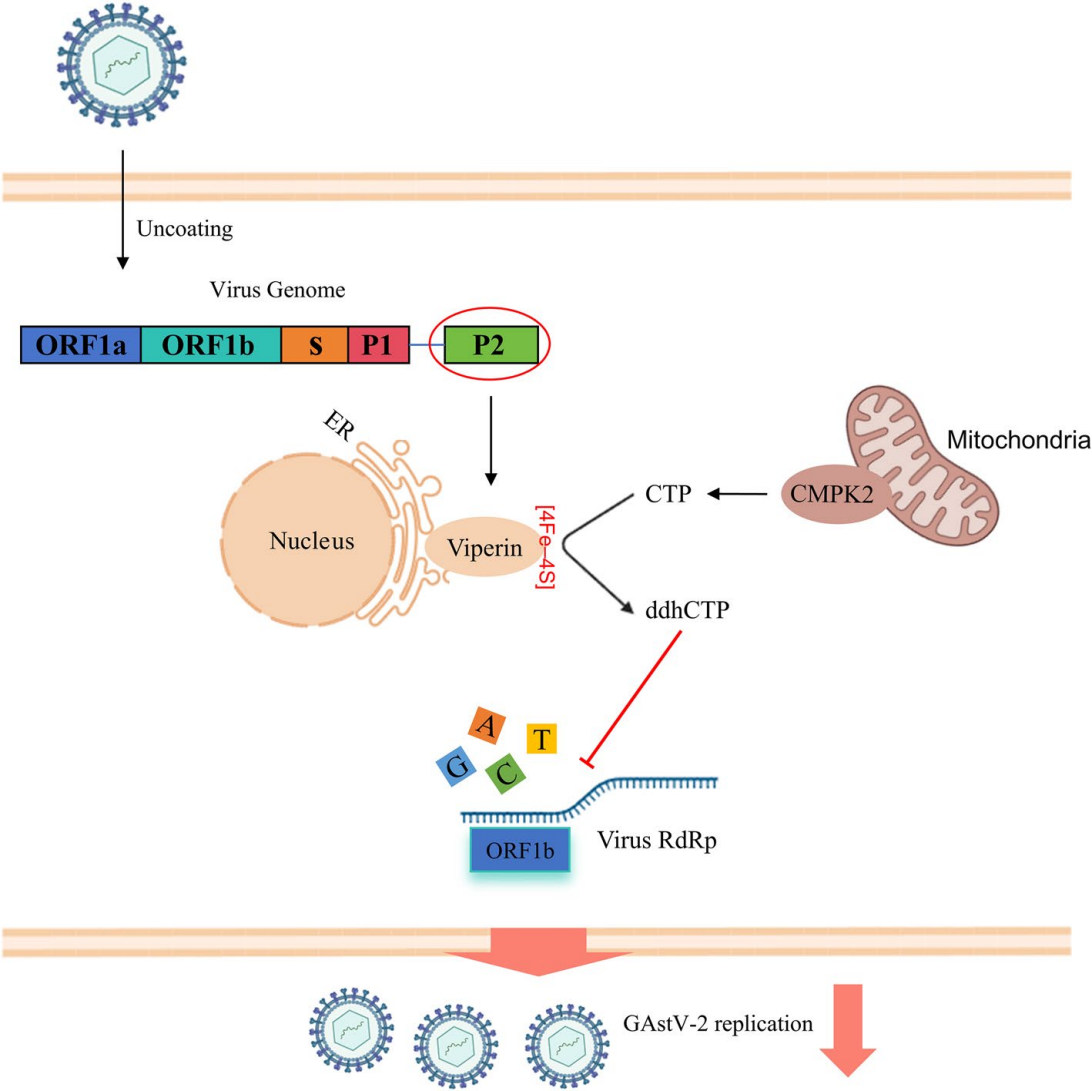
**Proposed mechanism by which Viperin restricts goose astrovirus type 2 replication through inhibition of viral RdRP activity.**

Our study demonstrates that Viperin is strongly upregulated during GAstV-2 infection and exerts significant antiviral effects. The SAM domain of Viperin is essential for this activity, mediating ddhCTP production and subsequent inhibition of viral RdRp (Figure 9). Exogenous ddhCTP administration effectively restricts GAstV-2 replication both in vitro and in vivo. These results establish Viperin as a key host defense factor against GAstV-2 and identify a potential new strategy for controlling GAstV-2 infection in geese.

## Data Availability

All data generated or analyzed during this study are included in this published article.
